# Structural Design and Assessing of Recombinantly Expressed African Swine Fever Virus p72 Trimer in *Saccharomyces cerevisiae*

**DOI:** 10.3389/fmicb.2022.802098

**Published:** 2022-06-14

**Authors:** Kaiwen Meng, Yueping Zhang, Qi Liu, Yangnan Huyan, Wenzhuang Zhu, Ye Xiang, Geng Meng

**Affiliations:** ^1^College of Veterinary Medicine, China Agricultural University, Beijing, China; ^2^School of Medicine, Tsinghua University, Beijing, China

**Keywords:** protein design, African swine fever virus, major capsid, p72 trimer, antigen

## Abstract

In an effort to control the outbreak of the African Swine Fever Virus (ASFV), there is an urgent need to develop an effective method to prevent the pandemic, including vaccines and diagnostic methods. The major capsid protein of ASFV p72 (B646L), which forms a trimer with each monomer adopting a double jelly roll fold, is the main component of the virus particle and major antigen of ASFV. Thus, the p72 protein may be considered an antigen candidate for vaccine and diagnostic development. However, the development of ASFV p72 trimer for the industry application, including veterinary usage, faces unavoidable challenges: firstly, the low cost of the antigen production is required in vaccine and diagnostic application; and, secondly, whether produced antigen folds in its native conformation. Here, based on the information provided by the atomic structure of p72, we have successfully performed rational mutagenesis on p72 trimers and expressed it in *Saccharomyces cerevisiae* with high yields. The cryo-EM structure of recombinant expressed p72 trimer is determined at 4.18 Å in resolution. The correlation coefficient between this structure and the ASFV virus structure is 0.77, suggesting a highly similar fold of this trimer with the native protein on the virus particle.

## Introduction

African swine fever (ASF), caused by the African swine fever virus (ASFV), is a highly contagious and fatal disease in domestic pigs (Parker et al., 1969; Thomson et al., 1980; Anderson et al., 1998). ASFV infection usually causes acute hemorrhagic fever in infected pigs, with a fatality rate as high as 100% (Blome et al., 2013; Pietschmann et al., 2015). Currently, there is no effective vaccine against ASFV. The ASFV outbreaks in China began in the summer of 2018 and have not been under control, so far (Zhou et al., 2018). The outbreak of ASF in China, the world's largest pork consumer, not only caused great harm to animal health but also brought major socio-economic consequences. Since the ASFV has not been eradicated, effective vaccines, and diagnostic methods need to be developed before the next pandemic.

The ASFV is the only member of the Asfarviridae family and belongs to the group of nucleocytoplasmic large DNA viruses (NCLDV). Previous studies have shown that the ASFV virion possesses a multilayered structure with a diameter of ~250–500 nm (Revilla et al., 2018; Wang et al., 2019), including a genome-containing nucleoid, a thick protein core-shell, an inner lipid membrane, an icosahedral protein capsid, and an outer lipid membrane (Salas and Andrés, 2013).

The major capsid protein p72 is the main component of the outmost icosahedral protein shell, accounting for 33% of the total mass of the virus (Carrascosa et al., 1984; García-Escudero et al., 1998). The p72 monomer contains two jelly roll fold domains that make up pseudo-hexameric capsomers in trimeric p72 and interact with the inner lipid membrane (Liu et al., 2019; Wang et al., 2019). Upon the pseudohexagonal base, a screw propeller-like cap is observed, which is composed of three β sheets blades inserted between jelly roll fold domains in each p72 monomer (Liu et al., 2019). Previous studies showed that p72 is one of the major antigens of infected pigs (Kollnberger et al., 2002), and is widely used as a marker for diagnosis of infection.

However, utilizing a full-length p72 trimer to establish a diagnostic kit or vaccine is complicated by the fact, that expression of p72 alone resulted in the formation of soluble aggregates rather than correctly folded and assembled into a p72 trimer (Liu et al., 2019). Efforts are being made to produce a well-folded recombinant p72 trimer. Liu et al. (2019) demonstrated that correctly folded and assembled p72 could be obtained when p72 and B602L were co-expressed, and the p72 trimer has high thermal stability. Moreover, several studies of rational protein engineering provide available suggestions for high yield production of stabilized p72 trimer. Rational protein engineering, which relies on electron maps in high-resolution, facilitates protein folding, and stability by introducing structural features into flexible regions. Multiple structural elements could contribute to protein stability in a predictable manner, including increased salt bridges, ion pairs, hydrogen bonds, and disulfide bonds (Balaji et al., 1989; Dill, 1990; Vogt et al., 1997; Kumar et al., 2000; McLellan et al., 2013; Porta et al., 2013; Joyce et al., 2016; Moore et al., 2017; Scott et al., 2017; Rutten et al., 2018; Yang et al., 2018). Moreover, introducing prolines in flexible regions can also increase rigidity (Balaji et al., 1989; Prajapati et al., 2007; Trevino et al., 2007; Pallesen et al., 2017; Hsieh et al., 2020; Wrapp et al., 2020). Recently, the proline trick has been successfully used to stabilize coronavirus spike proteins and increase the protein yields (Pallesen et al., 2017; Hsieh et al., 2020; Wrapp et al., 2020).

In this study, we performed a rational design based on the atomic structure of p72 to increase the yield and stability of the ASFV p72 trimer. By combining seven proline substitutions into a single construct, the trimeric state of p72 is stabilized, and the total yield of the p72 trimer could reach ~30 mg per liter of culture media. Structure validation by using cryo-electron microscopy (cryo-EM) confirmed that the proline substitutions do not disrupt the conformation of the p72 trimer, thus, preserving its antigenicity. This work will facilitate the production of p72 trimer for diagnostic kits and has even broader implications for ASFV subunit vaccine design.

## Materials and Methods

### *In Silico* Design Procedure

We used the cryo-EM structure of p72 (PDB ID: 6KU9) to design a stable trimer (Liu et al., 2019). The B-factor profile of the protein was analyzed. To determine the relative value of the contribution that each of the amino acids to the flexible regions, the relative B factor for each residue was calculated:


$$\begin{array}{l} {\text{B}}_{\text{relative}}=\text{B}–{\text{factor}/B}_{\text{average}} \end{array}$$

**Scheme 1:**
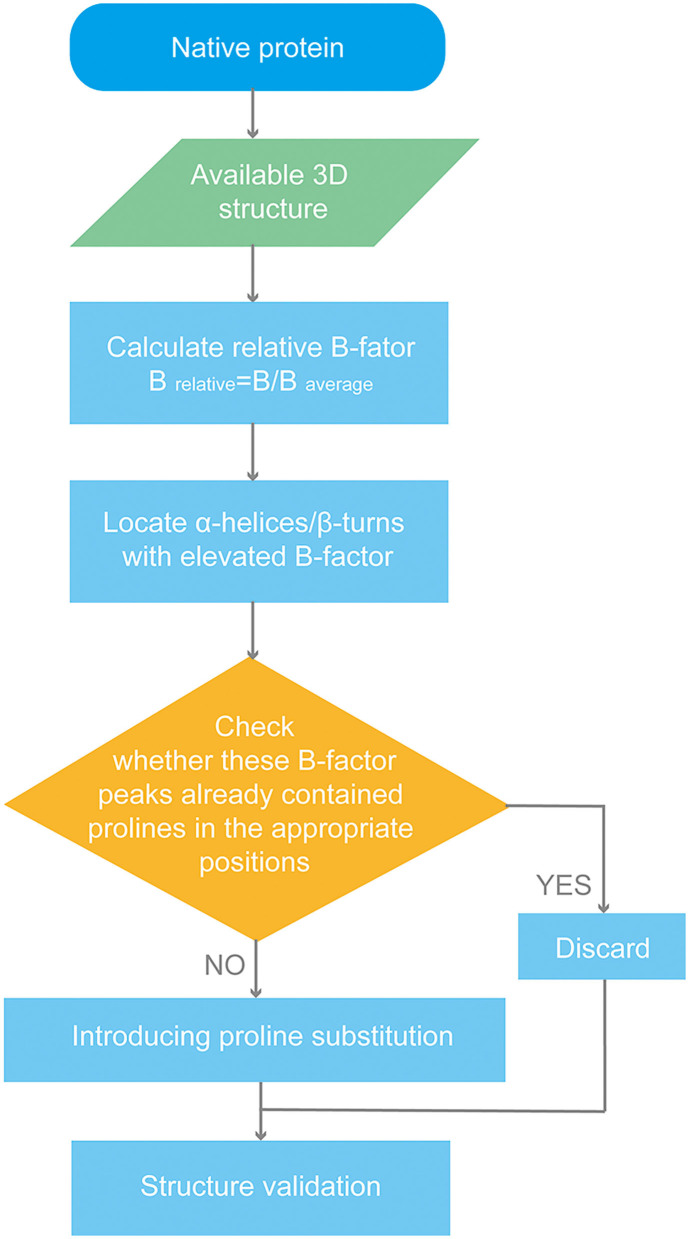
Flow chart of p72 rational design.

Considering p72 forms into trimers, B-factor is the mean value of the three available monomers' B-factors for each residue. B _average_ is the mean value of the B-factor for the p72 trimer. Peaks in the B-factor plot indicate flexible regions in p72. We introduced seven proline substitutions into flexible regions. The workflow is shown in Scheme 1.

### Analysis of p72 Variants Expression in *Saccharomyces cerevisiae*

The constructed expression cassette carrying gene encoding p72 (with Strep-tag) was integrated into yeast competent cells to give a new expression strain as previously described (Zhang et al., 2019). To compare the yields of each variant, the yeast strains expressing different variants were grown in a shake flask containing 4 ml of YPD and harvested cells, when O.D.600 reached about 1–2. Resuspended cell pellet in 250-μl NaOH/BME (2M NaOH, 10% BME), then, incubated on ice for 5 min. After spinning for 30 s at 14,000 rpm at room temperature, the precipitation was collected and resuspended by 250 μl of extraction buffer (500 mM of NaCl, 20 mM of Tris, pH8). The sample was spun again to collect precipitant, all precipitant was applied for sodium dodecyl sulfate-polyacrylamide gel electrophoresis (SDS-PAGE) analysis. The percentage of the expressed p72 in total cellular protein is determined by GelAnalyzer.

The solubility of recombinant native p72 and HeptaPro were compared by western blot. About 4-ml culture of each variant was spun to collect yeast cells. Resuspended precipitant with extraction buffer containing SDS, and disrupted yeast cell by bead beating. The supernatant and precipitant of cell disruption product were collected by centrifugation and applied for western blot analysis.

### Expression and Purification of p72 HeptaPro Trimers

Transformants integrated with HeptaPro expression cassette were selected on plates containing SC-Ura medium after incubation at 30°C for 2 days. The new strain was grown in a shake flask containing 50 mL of YPD, at 30°C overnight. Then, the overnight cultures of cells were inoculated in 1-L YPD. The culture was harvested by centrifugation at 6,000 rpm for 10 min when the O.D.600 reached about 3. Cell pellets were resuspended in 50 mL of Buffer W (IBA), followed by cell disruption by utilizing the homogenizing machine. The supernatant of cell disruption product was collected after centrifugation at 17,000 rpm for 90 min, and all supernatant was loaded onto a Strep-Tactin XT gravity-flow column (IBA). The collected eluents were analyzed with SDS-PAGE and the final elution was selected based on purity. The final pool was concentrated to >5 mg/mL using Merck-Millipore 30 kDa, 15 mL of concentrator. The BCA assay was performed on microtiter plates for both in-process and final product analysis to determine the protein concentration of p72.

### SE (Size Exclusion)-FPLC

The Size Exclusion-Fast Protein Liquid Chromatography (SE-FPLC) analysis of purified p72 was performed on an FPLC size exclusion chromatography column (GE healthcare, Superdex 200, 16/600) operated at a column temperature of 4°C on an AKTA system. The mobile phase consisted of 150 mM sodium chloride and 20 mM TRIS (pH 8.5) with a flow rate of 1 mL/min. The final pool was concentrated to >5 mg/mL using Merck-Millipore 30 kDa, 15 mL of concentrator.

### SDS-PAGE and Western Blot

For protein analysis, samples were diluted with 5 × Loading sample buffer, heated at 95°C for 10 min, and loaded on 10% SDS-PAGE gels. Gels were run at 140 V for 60 min in l × Tris-Glycine running buffer and stained with Coomassie Blue Stain solution. Following SDS-PAGE, proteins were transferred onto 0.2 pm nitrocellulose membrane (Pall) and blocked in 5% skim milk in TBS at 4°C overnight. Primary antibody at a 1:5,000 dilution of anti-strep antibody in 2% skim milk in TBST, was added and incubated for 1 h at room temperature. Membranes were washed with TBST (3 × for 10 min each) and added with secondary antibody 1:5,000 dilution of goat anti-mouse IgG-HRP in 2% skim milk (PBST) for incubation at room temperature for 1 h. Membranes were then washed again with TBST (3 × for 10 min each) and developed using TMB (3,3,5,5'-Tetramethylbenzidine, Sigma Aldrich) and hydrogen peroxide for imagining.

### Mass Spectrum Analysis

Following SDS- PAGE gels, the bands corresponding to p72 were collected, and mass spectrum (MS) analysis was performed by Mass Spectrum Laboratory, College of Biological Science, China Agricultural University.

### Negative-Stain Electron Microscopy

The p72 trimer particles were imaged by negative-stain electron microscopy (EM). About 5 μl of samples were loaded onto a pre-glow discharged carbon-coated EM grid (Beijing Xinxing Braim Technology Co., Ltd.), and then, stained with 2% p-phthalic acid (PTA) for 60 s. Micrographs were collected on a 120-kV FEI Tecnai Spirit microscope.

### Cryo-EM Data Collection and Image Processing

A pre-glow discharged 200 mesh Quantifoil grid (1.2 μm hole size) was used to load samples at 85% humidity and 4°C using a Vitrobot Mark III (FEI company). Cryo-EM images were collected using a Titan Krios electron microscope operating at 300 kV and equipped with a K3 Summit camera (Gatan). A full description of the data collection parameters can be found in Supplementary Table 1. Motion correction, contrast transfer function (CTF) estimation, and particle picking were performed in RELION. A total of 336,206 particles were imported into RELION for 2D classification, 3D reconstruction, and refinement. The final resolution determined by the gold standard Fourrier Shell Correlation (FSC) was 4.18 Å after postprocessing, and maps were visualized using UCSF Chimera.

### ELISA

Serum samples were collected from pigs that survived ASFV infection, recovered from the disease, or became carriers of ASFV. The blood samples were centrifuged at 1,200 × g for 15 min with the pellet removed, and serum samples were stored at −20°C. The enzyme-linked immunosorbent assay (ELISA) kits were established by our laboratory for the detection of antibodies against ASFV p72. Plates were read by Multiskan FC (ThermoFisher) at 450 nm.

Sensitivity was determined by a serum dilution series, and the results were compared with those detected by ELISA kits coated with p54 or p30. The specificity of the ELISA was verified with serum samples from pigs infected with viruses, as well as healthy controls. The reliability was demonstrated by testing 92 clinical serum samples and calculating the coincidence rate with the standard commercial ELISA kit (ID-Vet).

## Result

### B-Factor Guided Proline Substitutions in p72

To generate a stabilized p72 trimer expressed at high levels, we analyzed the p72 cryo-EM structure (PDB ID: 6KU9) and designed substitutions based upon knowledge of general protein stability principles. This strategy stabilized loops, β-turns, and helices by introducing proline in flexible regions.

**Figure 1:**
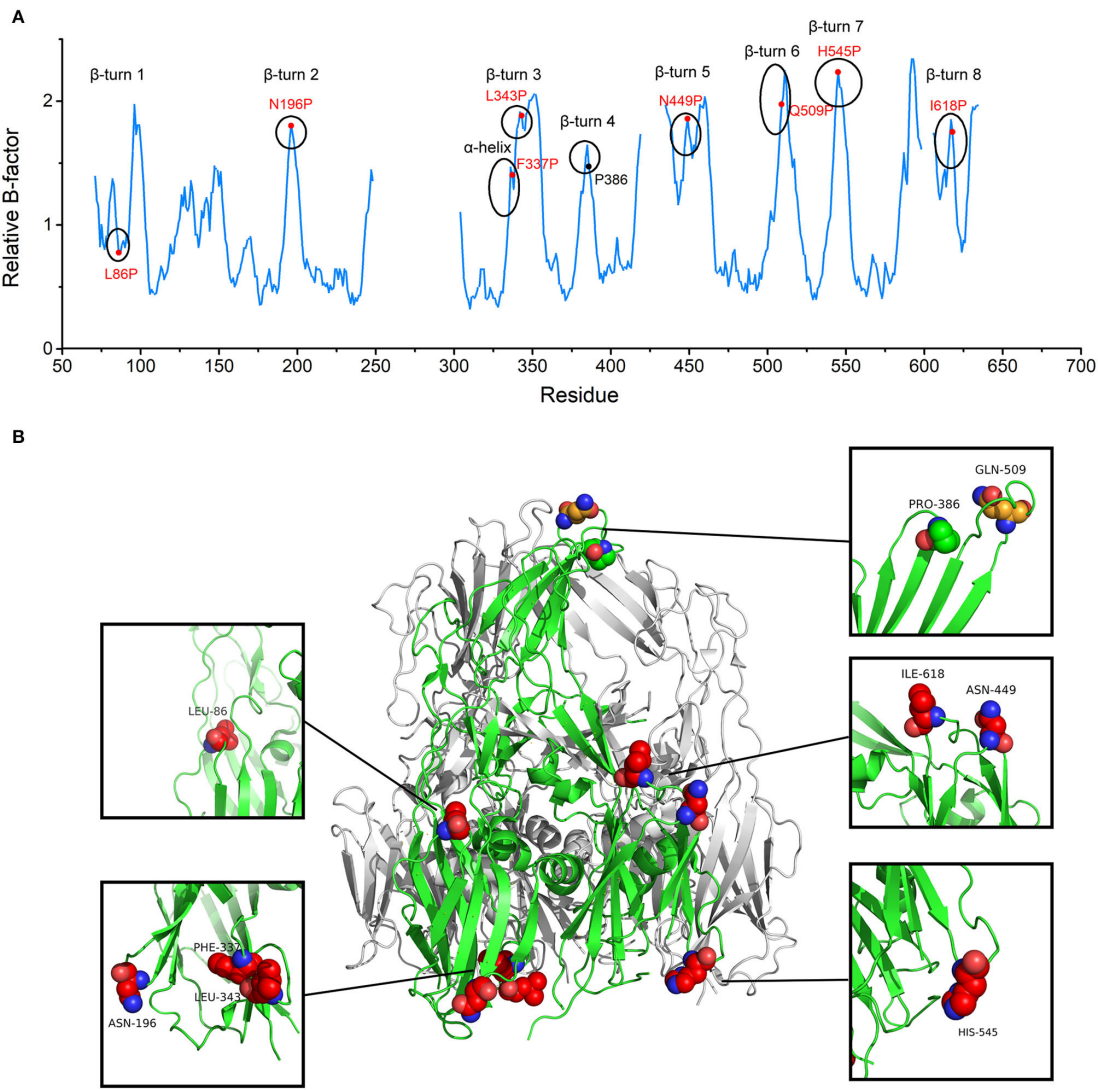
The target substitution sites for African Swine Fever Virus (ASFV) p72 trimer stabilization. **(A)** p72 relative B-factor/residue plotted against amino acid residue number. Each secondary structure element with elevated relative B-factor is marked with a circle. Red labels denote residues initially chosen for mutation and black label denotes already exists proline. **(B)** The side view of the trimeric ASFV p72 (PDB ID: 6KU9) ribbon diagram. One of the p72 is shown in green. Residues within β turn and α helix with elevated relative B-factor are shown as spheres. The finally determined substitution sites are shown as red spheres. The residue located on the most exposed top of the screw propeller-like cap and the already existed proline are shown as orange sphere and green sphere, respectively.

In accordance with the Proline rule, the peaks in the B-factor plot belonged to β-turns and α-helix. Peaks already contained prolines in the appropriate positions were discarded. Eight positions were screened for proline substitution based on their elevated B-factors about nearby residues, including L86, N196, F337, L343, N449, Q509, H545, and I618 (Figure 1 and Supplementary Figure 1). To preserve immunogenicity, Q509, a residue located on the most exposed top of the screw propeller-like cap, was not considered for substitution (Figure 1B). Initially, the seven selected proline mutations were combined into one seven-point mutant. Single-substitution variants and combination variants were also generated. The variant which produces soluble and stable protein was selected for further study.

### HeptaPro Resulted in Drastic Increases in Expression

The recombinant p72 was successfully produced in the *Saccharomyces cerevisiae* system and purified by Strep-Tactin affinity chromatography. Among all constructs, all variants with single substitution show no significant change in p72 yields (Supplementary Figure 2A). Two variants combing multiple substitutions were also generated, named Combo 1 and Combo 2. In combo 1 and 2, substitutions were introduced in the N-terminal jelly roll and C-terminal jelly roll, respectively. The result revealed that combos 1 and 2 produce a ~2-fold increase in protein yield compared with the recombinant native p72. Moreover, recombinant p72 with all seven proline substitutions, named as HeptaPro, exhibit a ~4-fold increase in protein yield, as well as higher solubility relative to the recombinant native p72 (Supplementary Figure 2). We were able to generate ~30 mg of HeptaPro from 1 L of culture media.

**Figure 2:**
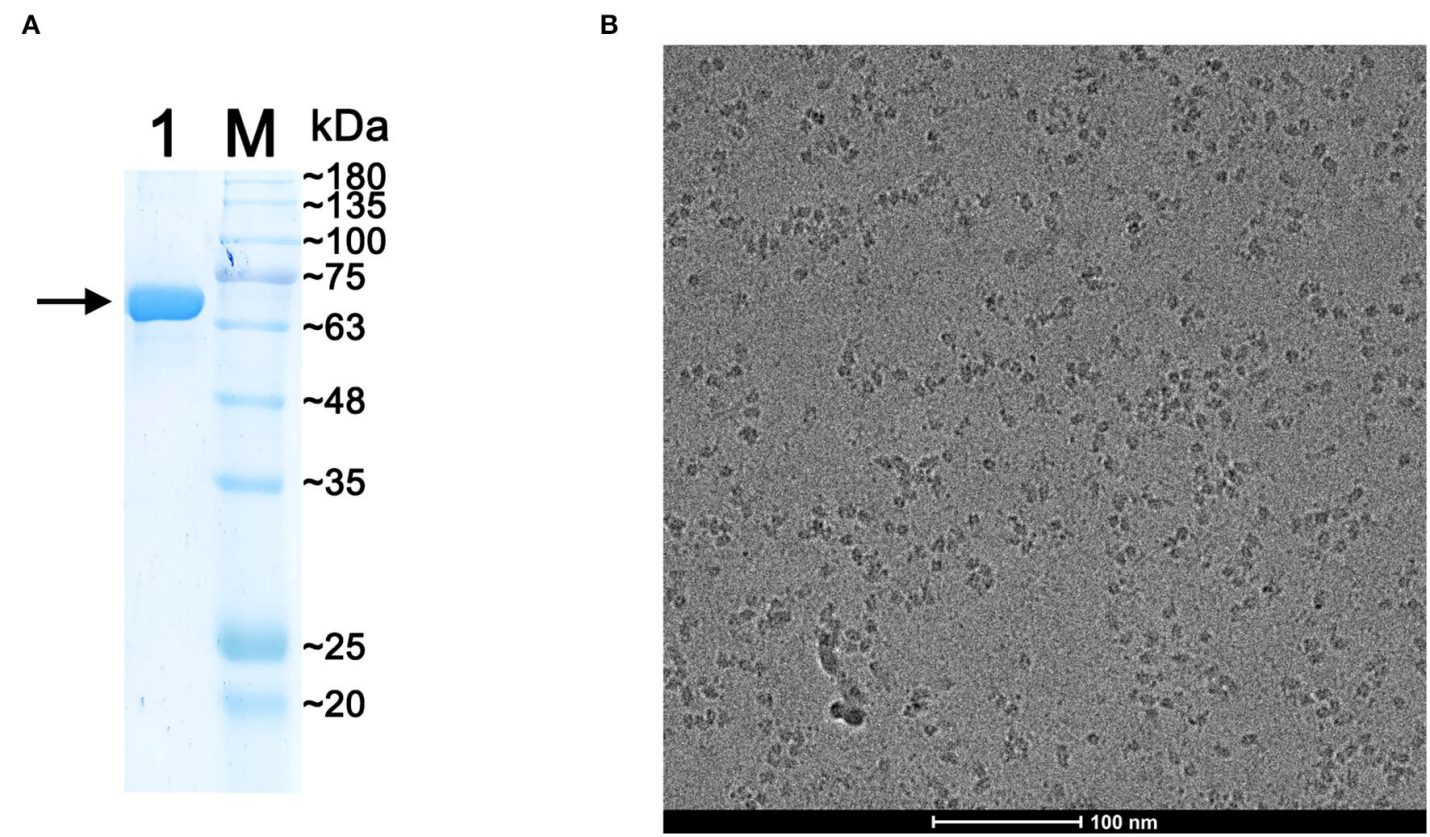
Preparation of the purified p72 sample. **(A)** Sodium dodecyl sulfate-polyacrylamide gel electrophoresis (SDS-PAGE) of purified p72, the arrow indicates the band of p72. **(B)** The Cryo-EM image of p72 trimer particles.

The expression of recombinant p72 was verified by western blot analysis (Supplementary Figure 2B), and the band was detected using an anti-strep antibody as described in materials and methods sections. Purified protein samples were analyzed by 10% SDS-PAGE, and gels were stained with Coomassie Blue (Figure 2A). The band corresponding to p72 was located at the expected molecular mass, approximately at 73 kDa. The p72 band was then analyzed by MS. The results of intact mass determination were consistent with the theoretical mass of the protein. The protein sequence coverage was above 52%: the first 38 N-terminal amino acids (MASGGAFCLIANDGKADKIILAQDLLNSRISNIKNVNK) of p72 determined by MS were consistent with the sequences reported earlier.

To verify the polymerization state of HeptaPro, we employed SE-FPLC analysis (pH 8.5). The result showed a monodisperse SE-FPLC peak corresponding to the molecular weight of the p72 trimer (Supplementary Figure 3), and no significant degradation was observed. We focused on this construct for additional structure validation.

### Cryo-EM Structure of ASFV p72 HeptaPro

**Figure 3:**
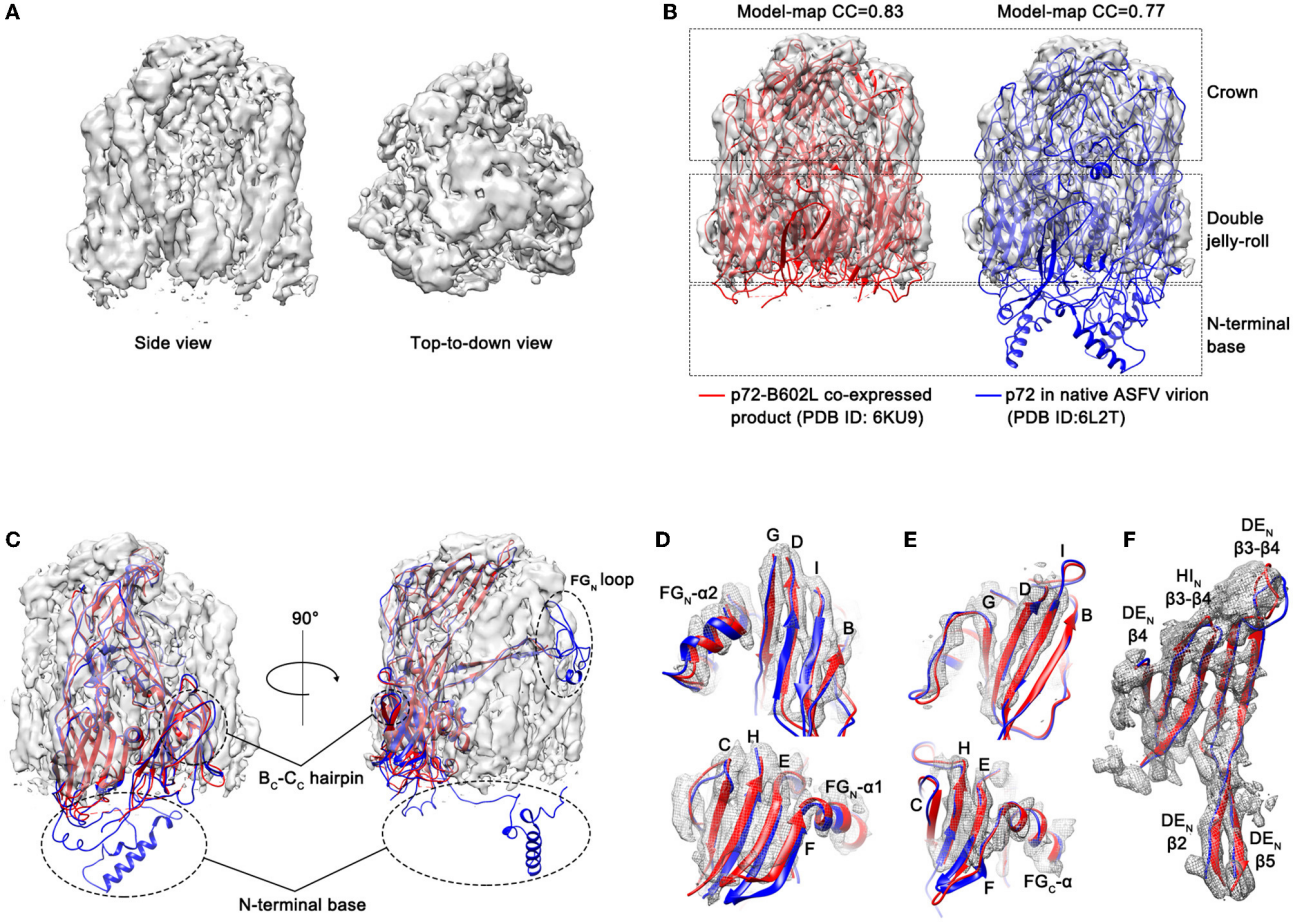
Electron microscopic observation results of p72 trimer. **(A)** Cryo-EM map of the yeast expressed p72 trimer. The map is visualized using UCSF Chimera at a contour level of 0.015. **(B)** The fitted p72 trimers (red ribbons, 6KU9; blue ribbons, 6L2T) in the cryo-EM map of the yeast expressed p72 trimer. The map is shown as semi-transparent solid surface. The model-map correlation coefficients (CC) are shown upon the structures. **(C)** The fitted p72 monomer in the cryo-EM map of HeptaPro. Dashed line frame highlights the disordered regions, including B_C_-C_C_ hairpin, FG_N_ loop and N-terminal base. **(D–F)** Fit of the p72 backbone into the cryo-EM density for the β sheets and the α helix of N-terminal jelly roll **(D)**, C-terminal jelly roll **(E)**, and crown domain **(F)**.

To confirm that the introduction of proline substitutions does not lead to any unintended conformational change, the purified ASFV p72 HeptaPro was then applied for electron microscope observation. The cryo-EM image of the purified HeptaPro sample is shown in Figure 2B. Then, we used cryo-EM single-particle 3D reconstruction to determine the structure of HeptaPro with a resolution of 4.18 Å (Figure 3A and Supplementary Figures 4, 5).

To evaluate the structural similarity between HeptaPro, p72-B602L co-expressed product (PDB ID: 6KU9, EMDB ID: 0776; Liu et al., 2019) and p72 in native ASFV virion (PDB ID: 6L2T, EMDB ID: 0814; Wang et al., 2019), both model-map correlation coefficient (CC) value and map-map CC value were calculated (Figure 3B and Supplementary Figure 6). The model-map CC between each PDB entry with its map, and the model-model CC between PDB entries 6KU9 and 6L2T were used as the reference value. The map-map CC value between HeptaPro and the previously solved p72 trimer densities ≥0.83. These results demonstrated that the conformation of HeptaPro resembles that of native p72 in an assembled virion.

The electron densities of some regions were missing in the HeptaPro, including the N-terminal base, the tip of the FG_N_ loop, and the B_C_-C_C_ hairpin (Figure 3C), which may cause by structural flexibility. And the quality of the cryo-EM map allows us to visualize most β sheets and α helices in the double jelly roll domain and crown domain (Figures 3D–F and Supplementary Table 2).

### HeptaPro Shows High Sensitivity and Specificity in Serological Diagnostic in Carrier Pigs

An indirect ELISA assay for the detection of ASFV p72 specific antibodies was developed using recombinant HeptaPro. This ELISA assay was applied to screen for specific p72 antibodies in serum samples from pigs that survived ASFV infection, recovered from the disease, or became ASFV carriers (carrier pigs). As presented in Supplementary Table 3, high levels of HeptaPro-specific antibodies were detected in all 6 carrier pigs.

Compared with p54 or p30 coated ELISA kits, HeptaPro-coated ELISA kits showed the highest sensitivity (Supplementary Figure 7). The antibody in serum could be detected even after 1:16,000 dilution when using HeptaPro as coated antigen. In addition, the HeptaPro-coated ELISA kit also showed great specificity and reliability (Supplementary Tables 4, 5). Overall, these data indicate that HeptaPro is a promising candidate for the development of diagnostic kits.

## Discussion

The major capsid protein p72 is the main component of the outmost icosahedral protein shell. So far, the immunogenicity of p72 has already been confirmed (Zsak et al., 1993; Neilan et al., 2004). Neilan et al. (2004) expressed a series of recombinant antigens including p72 through the baculovirus expression system. Also, the results of immunization and challenge experiments showed that all vaccinated animals died between 7 and 10 days post-infection (DPI), albeit the test group animals exhibited a 2-day delay in the onset of clinical disease and reduced viremia levels at 2 DPI. Their results indicated that the antibody induced by baculovirus expressed p72 may exert a certain neutralizing activity against ASFV, but still not efficient for protection against the challenges of virulent virus.

In previous studies, p30 was reckoned as a promising candidate for the development of serological diagnostics, based on serum antibody response evaluation of recombinant ASFV antigens (p30, p54, and p72; Giménez-Lirola et al., 2016). The comparison showed that his-tagged soluble p30 responses at DPI ≥ 12 were higher than that of recombinant p72 expressed in inclusion bodies (Giménez-Lirola et al., 2016). However, due to the laborious process of denaturation and renaturation, it cannot be concluded that the protein expressed in inclusion is still in its natural state. Besides, more researchers found that there was no significant difference in viral expression of p30 and p72 in ASFV-infected cells (Hubner et al., 2019; Oh et al., 2021). Collectively, the reactivity of p72 was under-evaluated.

Liu et al. (2019) expressed soluble p72 trimer with the aid of B602L for the first time. Their results showed that the correctly folded and assembled p72 could be obtained only when p72 and B602L were co-expressed, and expression of p72 alone resulted in the formation of soluble aggregates. Their study provides a basis for the application of stabilized p72 trimer in diagnostics. Since the nucleotides length of p72 and B602L are 1,941 and 2,101 bp, respectively, multi-copy plasmids with such large fragments are often genetically unstable and difficult to integrate correctly into a host chromosome. The rational design based on structural information is a promising solution to bypass the limitation of large fragments.

In this study, we reported an optimized, scalable, and reproducible process to produce the correctly folded trimeric p72 by rational design. By introducing proline, the HeptaPro was proven to have a conformation as a p72 trimer in native ASFV virion without B602L. Finally, we demonstrated that 30 mg of well-folded HeptaPro per liter of the medium can be obtained at a laboratory scale, lighting the way to industrial-level production to meet global demand for stabilized p72 trimer.

**Figure 4:**
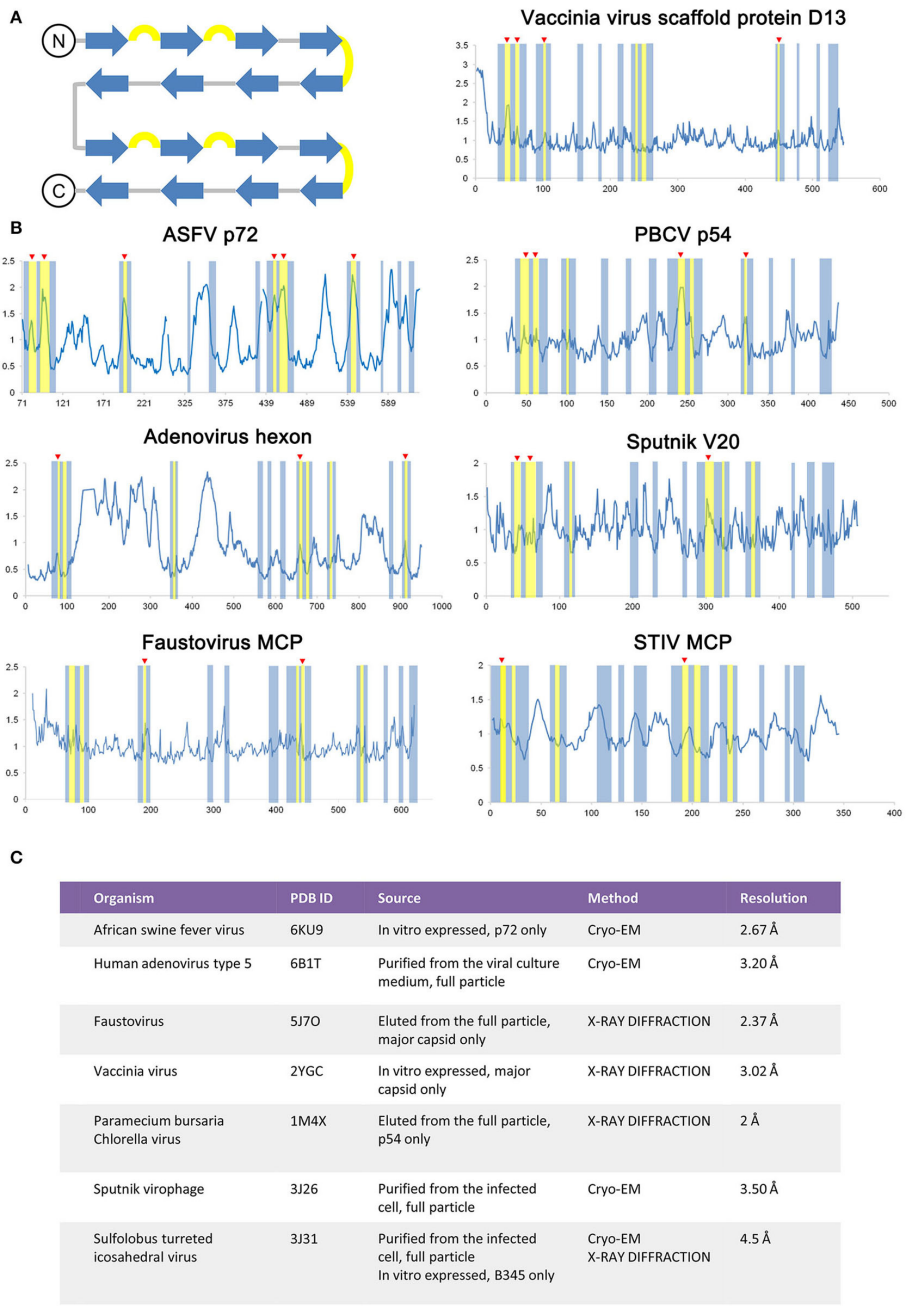
Comparison of the double jelly roll viral capsid or scaffold proteins. **(A)** The topology of a double jell-roll is shown, all insertions are left out. The β strands and β hairpin loops are colored blue and yellow, respectively. **(B)** The relative B-factor/residue plot against amino acid residue number of the double jelly roll viral capsid proteins and scaffold protein, including the ASFV p72 (PDB ID: 6KU9), adenovirus hexon (PDB ID: 6B1T), faustovirus MCP (PDB ID: 5J7O), Vaccinia virus scaffold protein D13 (PDB ID: 2YGC), *Paramecium bursaria* chlorella virus (PBCV) p54 (PDB ID: 1M4X), Sputnik V20 (PDB ID: 3J26), Sulfolobus turreted icosahedral virus (STIV) MCP (PDB ID: 3J31). The secondary structure region corresponding to double jelly roll is highlighted following the color rule in **(A)**. Secondary structure element with elevated relative B-factor is indicated with red triangle marks. Detailed information of each double jelly roll viral capsid protein and scaffold protein is shown in **(C)**.

As the ASFV p72 adopts a double jelly roll structure, other viral capsid proteins, and scaffold protein adopt a similar structure and were also examined for secondary structure-related B-factor variation (Figure 4). The relationship between the B-factor and structural rigidity has been reported in X-ray diffraction data analysis (Trueblood et al., 1996; Sun et al., 2019). Here, by comparing the variation of B-relative extracted from double jelly-roll proteins, the B-factor's elevation at β hairpin loops within jelly-roll barrels is observed in both crystal structures and Cryo-EM structures (Figure 4). Thus, it is rational to use the B-factor to identify the unstable regions in this case. Introducing proline is a reasonable solution for the stabilization of proteins adopted double jelly roll and may even improve their solubility and yields.

According to our study on the sera of carrier pigs, the results validated that the HeptaPro can be recognized by antibodies from the carrier, which implies that p72 may be an important neutralizing target in the process of ASFV immunization, and anti-p72 antibodies have the potential to neutralize the virulent virus. Furthermore, the high sensitivity and specificity demonstrated by HeptaPro-coated ELISA kits imply the importance of coated antigen conformation.

In conclusion, the result showed a high-yield method to produce a p72 trimer by rational design. The availability of this recombinant protein in pure form would facilitate the development of efficient ASFV subunit vaccines and diagnostics.

## Data Availability Statement

The datasets presented in this study can be found in online repositories. The names of the repository/repositories and accession number(s) can be found in the article/Supplementary Material.

## Author Contributions

GM conceived and designed the experiments. KM and YZ performed an expression experiment. KM, QL, YX, and GM determined the structure. WZ and YH performed the ELISA experiments. GM, KM, and YH wrote the manuscript. GM, YZ, and KM reviewed and edited the manuscript. All authors contributed to the article and approved the submitted version.

## Funding

This study was funded by the National Key Research and Development Program (2019YFC1604600) and the National Natural Science Foundation of Hebei province (19226631D).

## Conflict of Interest

The authors declare a patient (CN 202110522777.4) issued for the preparation method and the application of African Swine Fever Virus capsid protein p72 trimer.

## Publisher's Note

All claims expressed in this article are solely those of the authors and do not necessarily represent those of their affiliated organizations, or those of the publisher, the editors and the reviewers. Any product that may be evaluated in this article, or claim that may be made by its manufacturer, is not guaranteed or endorsed by the publisher.
