# Supplementary material for: Structural Design and Assessing of Recombinantly Expressed African Swine Fever Virus p72 Trimer in Saccharomyces cerevisiae
Source: Front Microbiol. 2022 Jun 14;13:802098. doi: 10.3389/fmicb.2022.802098 (PMC9239254; doi:10.3389/fmicb.2022.802098)
Supplement: Supplementary file 1 [file Data_Sheet_1.docx]

Supplementary Material


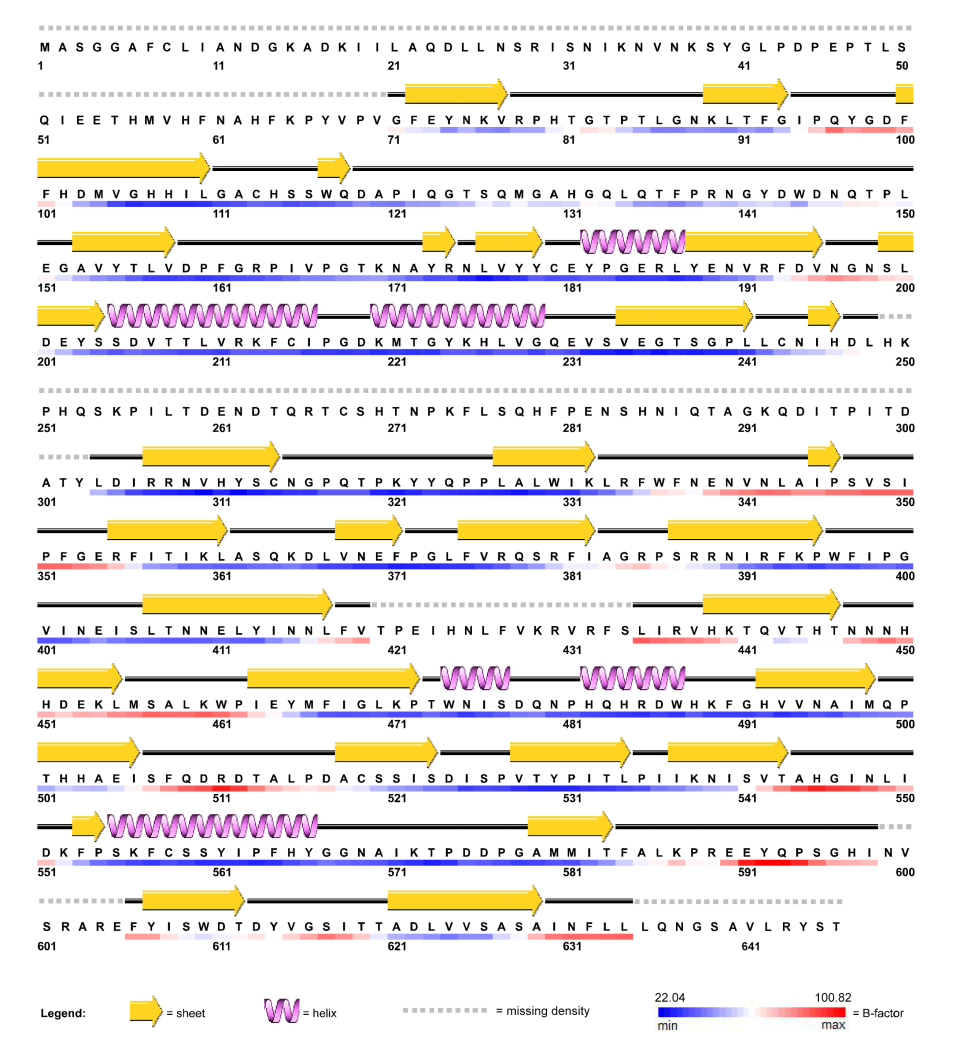


**Figure S1. Detailed secondary structure for the p72 (PDB ID: 6KU9), including original B-factors of each residue. All information is drawn directly from the PDB file.** The result was generated by Disulfide by Design 2.0[1-3].


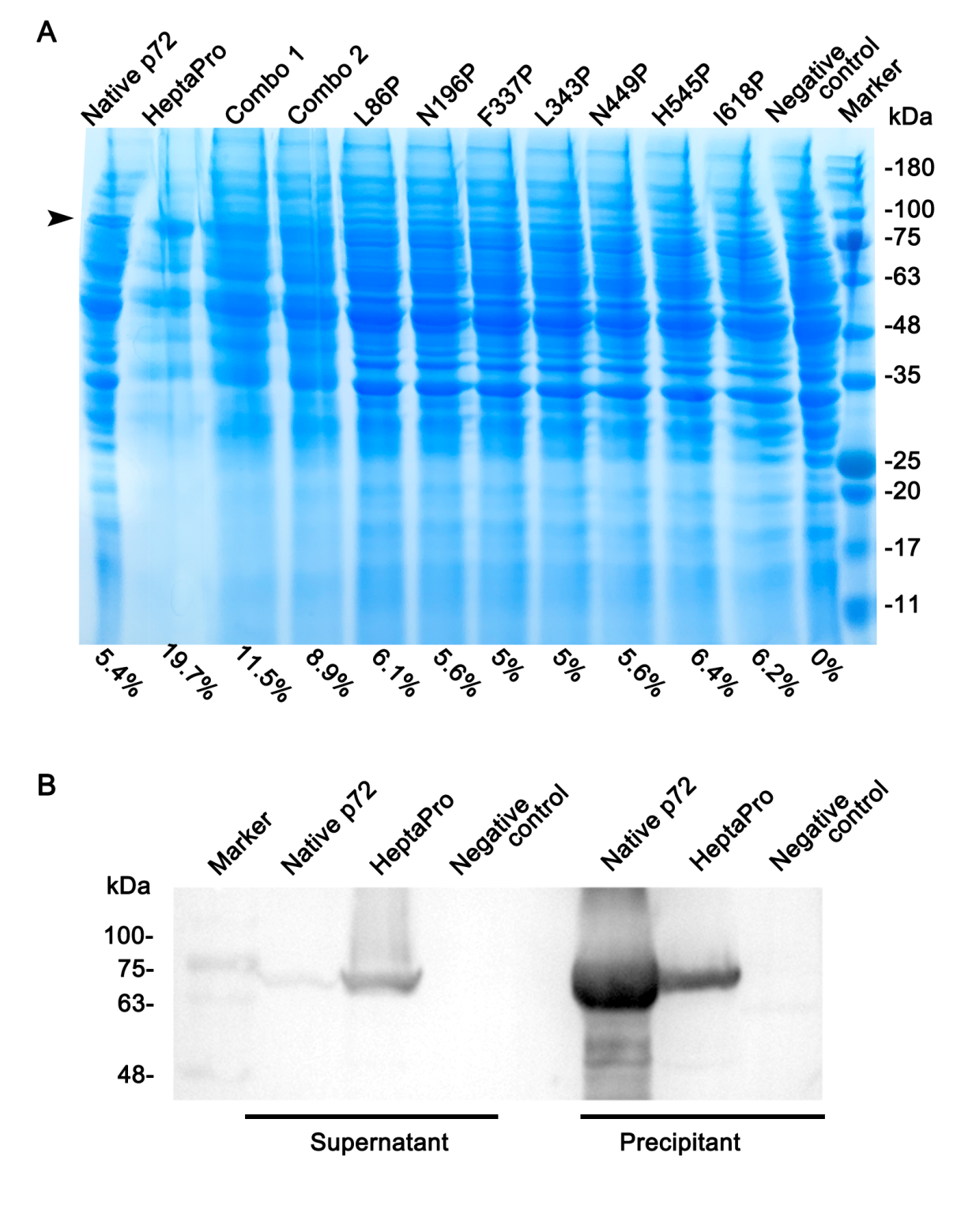


**Figure S2. Analysis of p72 expression.**

(A) SDS-PAGE analysis of p72 expression. The name of each variants is listed above the lane. The band corresponding to p72 is indicated by a black arrow. The percentage of the expressed p72 in total cellular protein is determined by GelAnalyzer, and listed below each lane. Combo 1 represents for variants combining multiple proline substitutions, including L86P, N196P, L343P, and F337P; and combo 2 represents for variants combing substitutions, including N449P, H545P and I618P.

(B)The solubility of native p72 and HeptaPro is compared by westernblot analysis. Both supernatant and precipitant after cell disruption are used for analyzing.


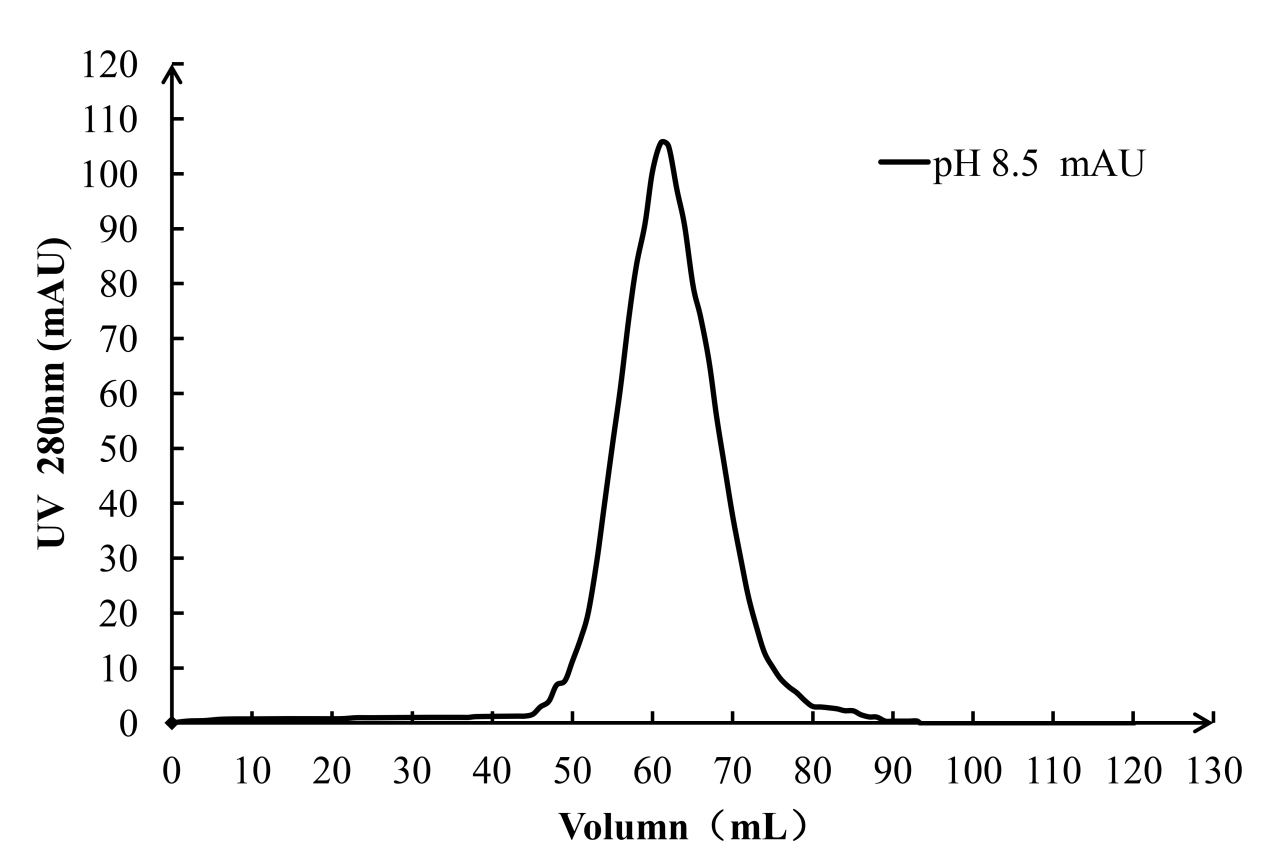


**Figure S3. Size exclusion FPLC analysis of p72 HeptaPro trimer.** The curve represents for the protein peak of p72 under pH8.5, and its peak position corresponds to ~210kda.


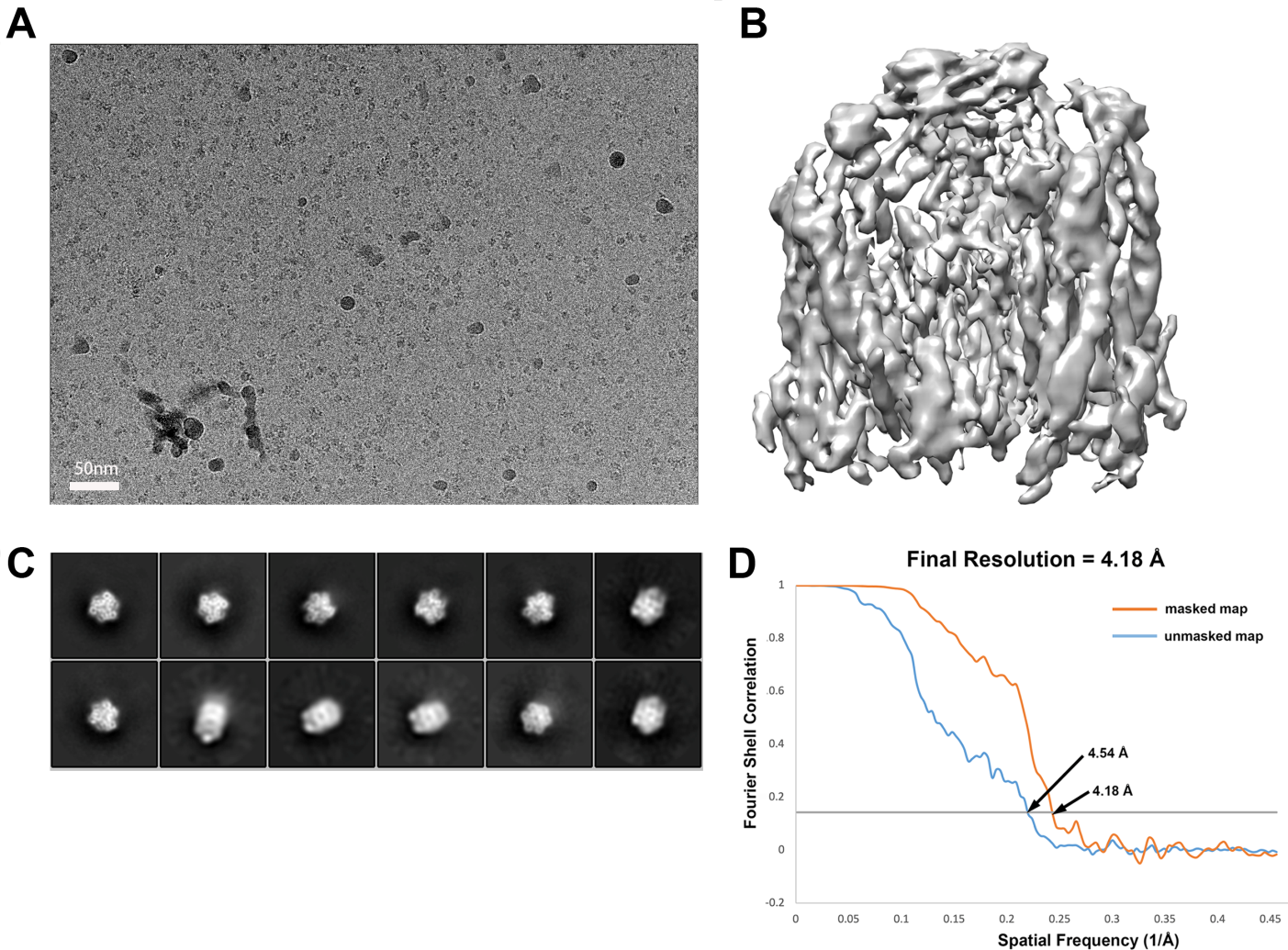


**Figure S4. CryoEM data and reconstruction results of HeptaPro.**

(A) A representative cryoEM micrograph. (B) EM density map of trimeric HeptaPro. (C) 2D classification. (D) The global Fourier shell correlation curves of the reconstruction.


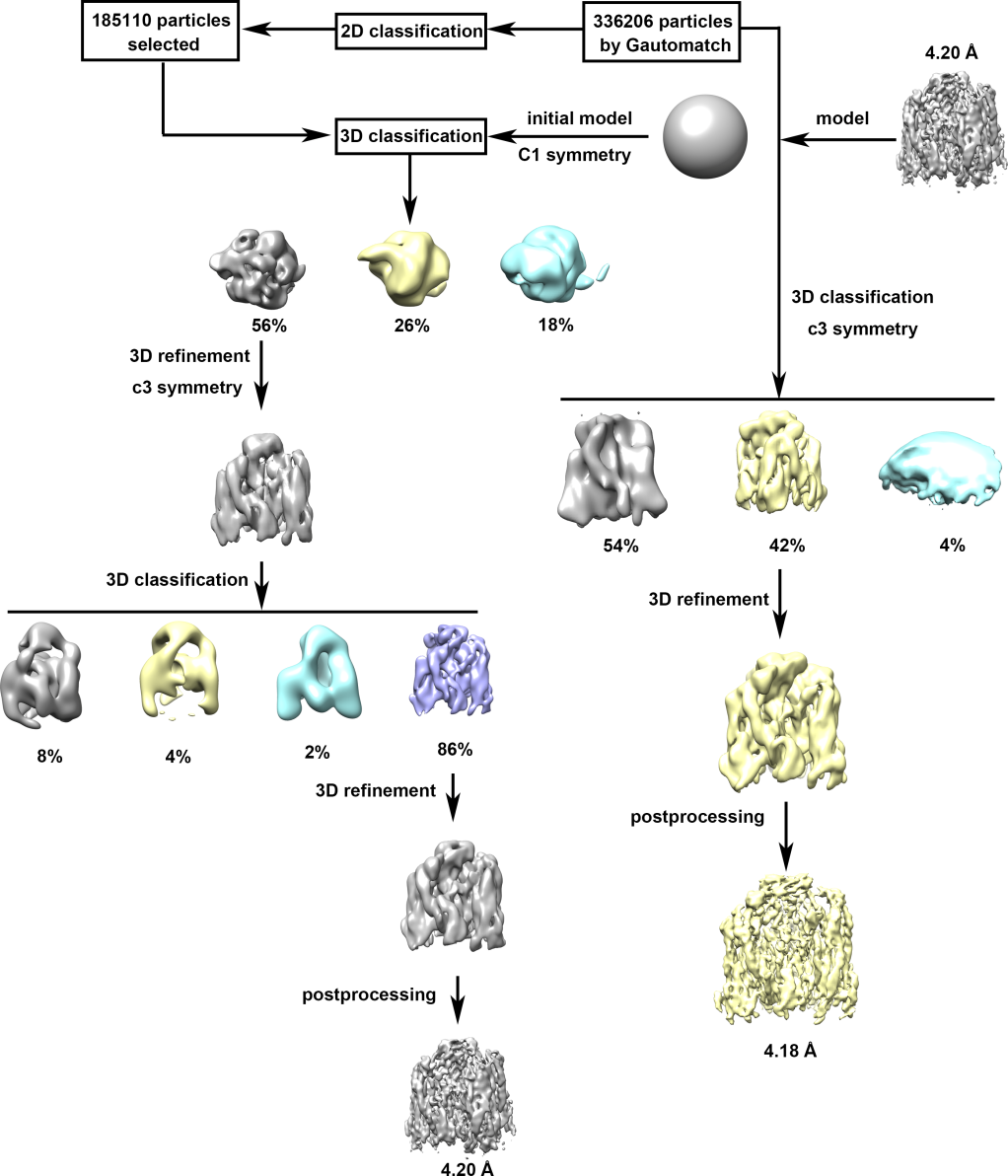


**Figure S5. Cryo-EM data processing workflow.**


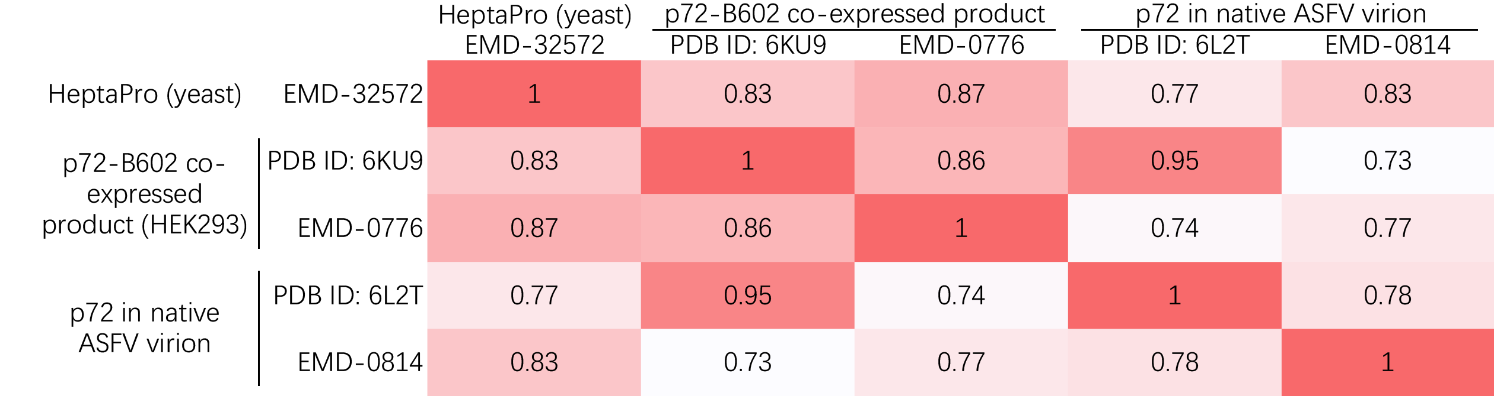


**Figure S6. Correlation coefficient matrix**


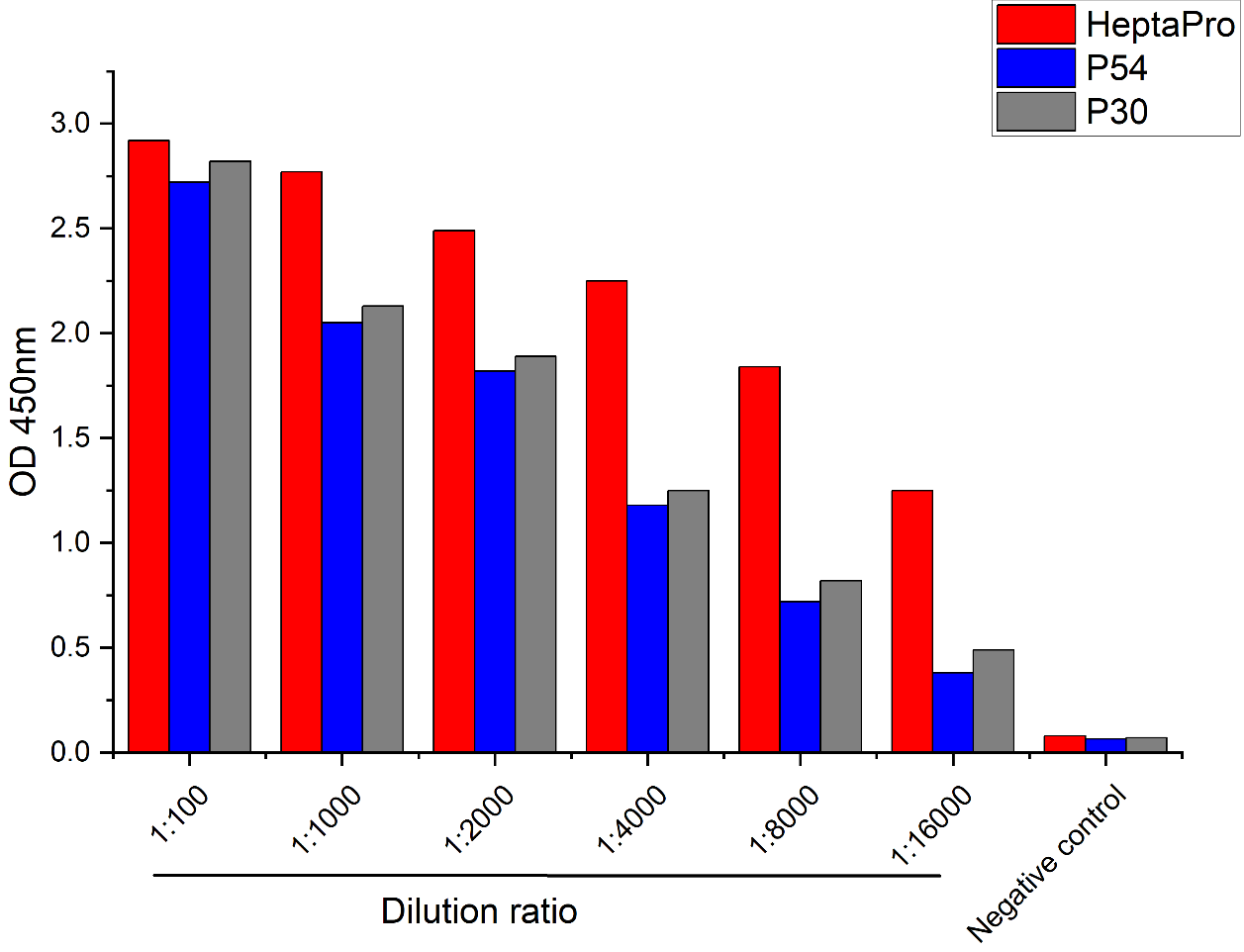


**Figure S7.Sensitivity of the ELISA kit coated with HeptaPro, P54 and P30**

**Table S1. Cryo-EM data collection and reconstruction statistics**

| Sample | p72 trimer |
| --- | --- |
| Acceleration voltage (keV) | 300 |
| Detector | K3 |
| Super-resolution pixel size (Å) | 0.54895 |
| Particles of final refinement | 143740 |
| Resolution (Å) | 4.18 |
| B-factor (Å2) | -176 |
| EMDB ID | EMD-32572 |

**Table S2. Local model-map correlation coefficient matrix between HeptaPro and PDB entries 6KU9 and 6L2T**

|  | | **6KU9** | **6L2T** |
| --- | --- | --- | --- |
| N-terminal jelly-roll | overall | 0.84 | 0.82 |
|  | BIDG sheets | 0.82 | 0.83 |
|  | CHEF sheets | 0.84 | 0.83 |
|  | α helix | 0.87 | 0.84 |
| C-terminal jelly-roll | overall | 0.8 | 0.79 |
|  | BIDG sheets | 0.78 | 0.77 |
|  | CHEF sheets | 0.79 | 0.81 |
|  | α helix | 0.85 | 0.85 |
| Crown | | 0.82 | 0.82 |

**Table S3. ELISA results of carrier pigs’ serum samples**

| **Pigs No.** | ***OD*_450nm_** | |
| --- | --- | --- |
|  | **1** | **2** |
| 1 | 2.11 | 2.049 |
| 2 | 1.778 | 1.79 |
| 3 | 1.808 | 1.721 |
| 4 | 1.843 | 1.767 |
| 5 | 1.741 | 1.731 |
| 6 | 1.747 | 1.696 |
| 7 (Healthy pig) | 0.085 | 0.084 |
| 8 (Healthy pig) | 0.042 | 0.043 |

**Table S4. Specificity of the HeptaPro coated ELISA kit for the detection of ASFV antibodies**

| **Sample** | ***OD*_450nm_** |
| --- | --- |
| ASFV antibody positive serum | 2.92 |
| Porcine circovirus antibody positive serum | 0.083 |
| Porcine reproductive and respiratory syndrome virus antibody positive serum | 0.075 |
| Porcine pseudorabies virus antibody positive serum | 0.072 |
| Classical swine fever virus antibody positive serum | 0.092 |
| Negative control | 0.079 |

**Table S5. Results of the HeptaPro coated ELISA kit and commercial ELISA.**

| **Method** | **Detection rate** | | | **Coincidence rate** | | | | **Overall coincidence rate** |
| --- | --- | --- | --- | --- | --- | --- | --- | --- |
|  | **＋** | **－** | | **＋** | | **－** | |  |
| **HeptaPro coated ELISA kit** | 65.2%  (60/92) | | 34.8%  (32/92) | | 100%  (57/57) | | 91.4%  (32/35) | 96.7%  (89/92) |
| **ID Vet ELISA** | 61.9%  (57/92) | | 38.1%  (35/92) | |  |  |  |  |

Reference:

1. Craig, D.B. and Dombkowski A.A. Disulfide by Design 2.0: a web-based tool for disulfide engineering in proteins. BMC Bioinformatics. 2013 Dec 1;14:346. DOI: 10.1186/1471-2105-14-346 PMID: 24289175
2. Dombkowski, A.A. Disulfide by Design: A computational method for the rational design of disulfide bonds in proteins. Bioinformatics. 2003 Sep 22; 19(14):1852-3. PMID: 14512360
3. Dombkowski, A.A., Sultana, K.Z., and Craig, D.B. Protein disulfide engineering. FEBS Letters. 2014 Jan 21;588(2):206-12. DOI: 10.1016/j.febslet.2013.11.024 PMID: 24291258
